# Microbial characterization of the Japanese traditional pickle senmaizuke produced by two different manufacturing processes

**DOI:** 10.1002/fsn3.2419

**Published:** 2021-06-19

**Authors:** Hiroyuki Iguchi

**Affiliations:** ^1^ Department of Agriculture and Food Technology Faculty of Bioenvironmental Sciences Kyoto University of Advanced Science Kameoka Japan

**Keywords:** lactic acid bacteria, pickles, turnip, vinegar, yeast

## Abstract

Senmaizuke, a traditional turnip pickle, prepared in Kyoto, Japan, is produced via two types of manufacturing processes: with and without vinegar for fermentation. The aim of this study was to reveal the microbial community and growth behavior in the products and manufacturing processes of two types of senmaizuke. Microbial growth analysis of commercial senmaizuke products showed that both types harbored 10^2^–10^8^ colony forming units (CFU)/g of lactic acid bacteria (LAB) and 10^2^–10^4^ of yeast. The fermented‐type products showed successive growth of LAB during the pickling and ripening processes, whereas LAB in vinegar‐type products showed growth only at the preliminary pickling step before vinegar addition; however, the bacteria were viable at the ripening step. LAB in the vinegar‐type products showed growth when the pH of the pickle was neutralized, indicating that acidification via vinegar retards LAB growth. Metagenomic sequencing showed that the fermented‐type products harbored *Lactobacillus, Leuconostoc*, and other halophilic and psychrophilic bacteria, with a higher bacterial diversity than in the vinegar‐type products. In the vinegar‐type products, *Lactobacillus*, *Leuconostoc*, or both were predominant. Culture tests using LAB isolates and turnip medium suggested that a change in the dominance of *Leuconostoc* to *Lactobacillus* members observed in the fermented‐type products during pickling and ripening processes was attributed to the low pH sensitivity of *Leuconostoc* as well as a relatively long lag phase of growth for adapting to the pickling environment. The findings of this study will be useful for the appropriate quality control and assurance procedure of senmaizuke.

## Graphical abstract

Senmaizuke, a traditional turnip pickle in Japan, is produced via two types of manufacturing processes: with and without vinegar for fermentation. Bacteria and yeasts in senmaizuke were analyzed via isolation and culturing as well as metagenomic sequencing. This study presents the first knowledge on the microbial community and growth behavior in the products and manufacturing processes of two types of senmaizuke.

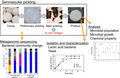

## 1 INTRODUCTION

Kyoto was considered the center of Japanese cuisine until the nineteenth century, and several cuisines and food materials including pickles have been developed in Kyoto. Shibazuke, suguki, and senmaizuke are “three major pickles in Kyoto.” Shibazuke and suguki are vegetable pickles fermented with lactic acid bacteria (LAB) (Shinagawa et al., 1997; Waki et al., 2014); nonfermented shibazuke that is prepared by soaking eggplants in a seasoning liquid is relatively more popular at present. Senmaizuke is prepared by soaking sliced turnips in a seasoning containing rice vinegar (hereafter called vinegar‐type senmaizuke) (Hiraga et al., 2008), and thus is considered a nonfermented pickle. However, senmaizuke was previously prepared via microbial fermentation without using vinegar (fermented‐type senmaizuke), which is now produced by factories in limited quantities.

Senmaizuke is produced during the winter season via two steps in a pickling process. In the preliminary pickling step, a large sliced turnip (Shogoin turnip; approximately 2–3 mm thick) is pickled using salts for a few days. During the main step, the turnip is pickled in a new seasoning containing salts, sugar, and vinegar together with kelp for a few days. Ripening may occur after packaging to a certain extent. The main difference between the vinegar‐type and fermented‐type senmaizuke is the use of vinegar at the main pickling step.

Vinegar can repress microbial growth in pickles (Adams, 1998); however, its relatively low concentration used for preparing vinegar‐type senmaizuke (approximately 0.3% acidity) may allow microbial growth, which can affect the flavor and shelf‐life (spoilage). Microbial control is an important process for maintaining the quality of the product including its flavor, especially for the fermented‐type senmaizuke; the addition of vinegar and other ingredients can impart a flavor to the vinegar‐type senmaizuke. However, the fermentation process of the fermented‐type senmaizuke, which involves a traditional and rarely used manufacturing method, has not been characterized at present. Moreover, the effects of vinegar on microbial growth and composition during the pickling and ripening processes of senmaizuke are poorly understood.

Lactic acid bacteria of the genera *Leuconostoc*, *Lactobacillus*, *Lactococcus*, *Weissella,* and *Pediococcus* are primarily involved in vegetable pickle fermentation (Swain et al., 2014; Tamang et al., 2016). The microbial composition as well as changes in the microbial community that occur via the pickling process is specific to each pickle and depends on the chemical (pH, osmotic pressure, antimicrobial agents), physical (vegetable structure for habitation and nutritional access), environmental (temperature, oxygen), microbiological (microbial source and competition), and nutritional conditions of the pickling process (Hammes & Hertel, 2006). The predominant LAB in kimchi includes members of the genera *Leuconostoc*, *Lactobacillus,* and *Weissella* (Patra et al., 2016), whereas those in sauerkraut are members of *Leuconostoc*, *Lactobacillus,* and *Pediococcus* (Wiander, 2016). In sauerkraut fermentation, the acidification of pickled cabbage generates conditions ideal for the growth of *Lactobacillus* and not *Leuconostoc* (Wiander, 2016). Fermentation of vegetable pickles at low temperature (10°C) enriches the psychrophilic LAB (Eom et al., 2007).

In this study, I aimed to determine the microbial characteristics of the traditional turnip pickle senmaizuke produced via two different manufacturing processes, with and without vinegar for fermentation. I analyzed the fermented‐type and vinegar‐type senmaizuke from one factory, and further evaluated a few commercial senmaizuke products obtained from other factories to identify the characteristics specific to senmaizuke. Analysis of the LAB and yeast strains isolated from senmaizuke further insights into the growth behavior of microbes present in senmaizuke.

## 2 MATERIALS AND METHODS

### 2.1 Culture conditions

Lactic acid bacteria was cultivated on MRS medium (BD) at 30°C. Calcium carbonate (0.5%, w/v) was added to the agar medium for detecting bacteria that produced lactic acid. Cycloheximide (50 µg/ml) was added to the medium for repressing fungal growth. The cultured media were incubated in a closed box containing an anaeropack (Mitsubishi Gas Chemical). Yeasts were cultivated at 30°C on potato dextrose agar (PDA; Nissui Pharmaceutical), or YPD liquid medium with shaking. Chloramphenicol (20 µg/ml) was added to the medium for inhibiting bacterial growth.

### 2.2 Senmaizuke sample

All products used were produced at Kyoto prefecture, Japan. Products A and B produced in the same factory were obtained from Shodai Kamekura company. Products C to K (each produced by a different company) were purchased at a local store, transported in ice to the laboratory, and analyzed on the same day. For analysis over a course of time, the products were stored at 4°C until analysis (herein referred to ripening process). Raw materials of the products are listed in Table S1. In terms of product form, the pickled turnips were packed in a sealed plastic bag containing pickled juice, except for product C that was packed in a non‐sealed plastic bag without pickled juice.

The chemical properties of the pickled juice were analyzed. LAQUAtwin compact meters (Horiba) were used to measure pH and salt concentration, and a refractometer (PAL‐1; Atago) was used to measure Brix.

### 2.3 Microbial population in senmaizuke

One slice of senmaizuke sample and 100 ml of saline (0.9% NaCl) solution were added to a plastic bag, followed by homogenization for 2 min using a stomacher (Pro‐media SH‐IIM; ELMEX). The homogenized solution was diluted with phosphate‐buffered saline and spread on MRS and PDA plates. After cultivation in the condition as described above, the number of colonies was counted to evaluate colony forming units (CFU) per wet weight g of senmaizuke. A single colony was picked up for the isolation of LAB and yeast.

### 2.4 Phylogenetic analysis

The 16S rRNA genes of the LAB isolates and the 26S rRNA genes of the yeast isolates were amplified via PCR using the primer sets 27f‐1492r and NL1‐NL4, respectively (O’Donell, 1989; Weisburg et al., 1991). Sanger sequencing using the 27f and NL1 primer provided approximately 650 bp and 500 bp sequences, respectively. These sequences were analyzed using the Blast program to identify related species. A phylogenetic tree of the 16S rRNA gene sequences was constructed using the ClustalW program (DDBJ, Shizuoka, Japan), by applying the Neighbor‐Joining method for a matrix of pairwise genetic distances for clustering. The sequences obtained in this study were deposited in DDBJ under accession number LC616606–LC616656.

### 2.5 Metagenomic analysis

Bacterial cells were collected from the pickled juice of senmaizuke samples via centrifugation. The cells were disrupted using zirconia beads, and genomic DNA was extracted and purified using the NucleoSpin Microbial DNA kit (TakaraBio). Sequencing analysis using an Illumina MiSeq platform was conducted by Bioengineering Lab. Co., Ltd. (Kanagawa, Japan). The V3/V4 region of the 16S rRNA gene was targeted. The sequences derived from plant chloroplast were excluded in the data analysis. The Shannon index (microbial diversity) was calculated using a previously described equation (Spellerberg & Fedor, 2003).

### 2.6 Pure culture and co‐culture experiments

To prepare the turnip medium, a raw turnip and an equal weight of water were homogenized using a food mixer. The homogenate was treated at 105°C for 1 min using an autoclave. The homogenate was filtered using a nonwoven fabric, and the extracted juice was mixed with sucrose (Brix 15%) and sodium chloride (salt meter 2%), after which the pH was adjusted using acetic acid. The turnip medium was treated at 105°C for 1 min for sterilization. The sterilization was confirmed by the absence of microbial growth on R2A agar (Nihon Pharmaceutical) and tryptic soy agar (BD).

The LAB and yeast strains were cultivated in liquid MRS and liquid YPD medium, respectively. The cell concentrations were modulated based on OD_600_ value, and the adequate quantity was inoculated into 30 ml of the turnip medium (in a capped polypropylene tube), which was incubated at 8°C. The CFUs of the LAB and yeasts in the culture medium were analyzed overtime via spread plating. Colonies of LAB strains, SL8 and SL35, were discriminated based on the colony color (white and yellow, respectively). LAB and yeast strains were discriminated using antibiotics.

## 3 RESULTS

### 3.1 General characteristics of senmaizuke commercial products

**FIGURE 1:**
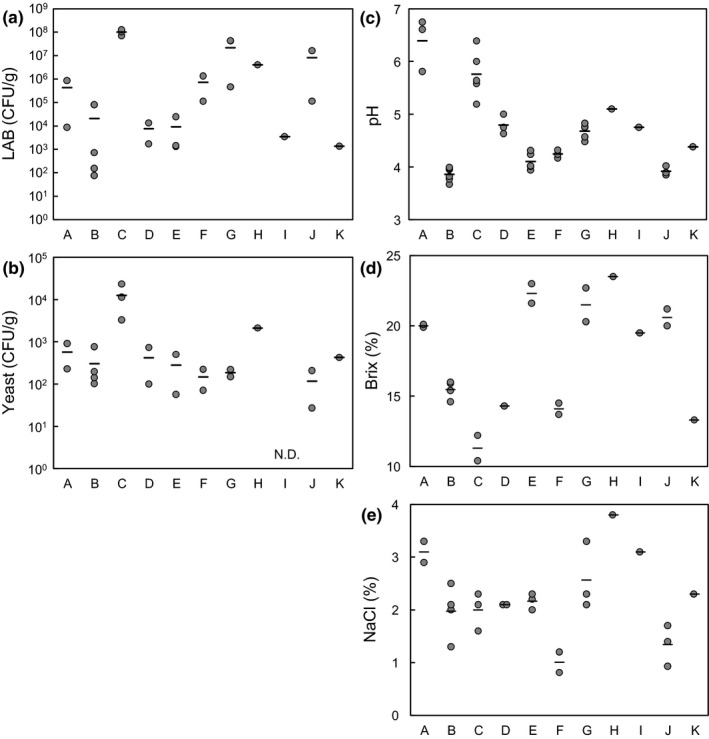
Microbial and chemical characteristics of commercial senmaizuke products. (a) LAB population, (b) yeast population, (c) pH, (d) Brix, and (e) NaCl concentration of products A‐ K. Plots represent the raw data points, and bars represent the average values (*N* = 1–6). Analyzed samples were from different lots (different production date or factory)

The microbial and chemical characteristics of the senmaizuke products from ten companies are shown in Figure 1. Products A and C were fermented‐type senmaizuke, whereas the other products were vinegar‐type senmaizuke. Product C contained the highest microbial load of LAB and yeasts among the tested samples, whereas the microbial load of product A was in the middle range. Few vinegar‐type senmaizuke products (G, H, and J) contained high LAB populations. In total, the population of LAB was between 10^2^ and 10^8^ CFU/g, and that of yeast was approximately 10^2^–10^4^ CFU/g in senmaizuke products. A relatively high pH (approximately pH6) was a common characteristic of the fermented‐type senmaizuke products. The pH of the vinegar‐type products was between 4 and 5. The values of Brix and NaCl varied between products, probably due to the intended flavors of each product.

### 3.2 Change in microbial growth behavior in senmaizuke during pickling and ripening processes

**FIGURE 2:**
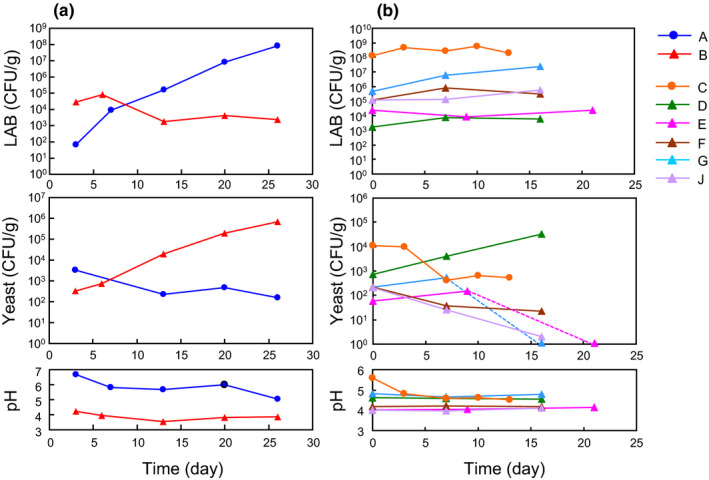
Changes in microbial population and pH during the pickling and ripening processes of senmaizuke. (a) Day 0 represents the beginning of production. The first plot represents the end of preliminary pickling, and the second plot represents the end of the main pickling, followed by packaging. (b) Day 0 represents the day when the product was purchased. Dotted line indicates below detection limit (approximately <5 CFU/g)

I studied the changes in microbial population in products A and B during the pickling and ripening processes (Figure 2a). In product A, the LAB population was 10^2^ CFU/g at the end of the preliminary pickling step (day 3), which then was continued to increase during the main pickling and ripening processes, concomitant with a slight decrease in pH. The yeast population was the highest at the end of the preliminary pickling step, and then it gradually decreased. In contrast, the yeast population in product B continued to increase during the pickling and ripening processes, whereas a significant increase in LAB population only occurred during preliminary pickling. LAB was not detected from raw turnip samples by direct spreading on agar medium. These results indicated that vinegar could have affected the growth behavior of LAB and yeast in senmaizuke.

Analysis of the other senmaizuke products during the ripening process was performed (Figure 2b). The vinegar‐type senmaizuke products showed an almost steady LAB population; however, an increase in LAB population over the tested period was observed in product G. The yeast population of the vinegar‐type senmaizuke decreased except in product D, in which the yeast population increased by 50‐fold in a period of 16 days. The relatively higher pH in products D and G (around pH5) were possibly associated with the microbial growth. Neither the population of LAB nor yeast was increased in fermented‐type product C, and the reason was assumed to be the relatively high populations at day 0. Altogether, the vinegar‐type senmaizuke may be characterized by a steady population of LAB at the ripening step.

### 3.3 Bacterial community composition in senmaizuke

**FIGURE 3:**
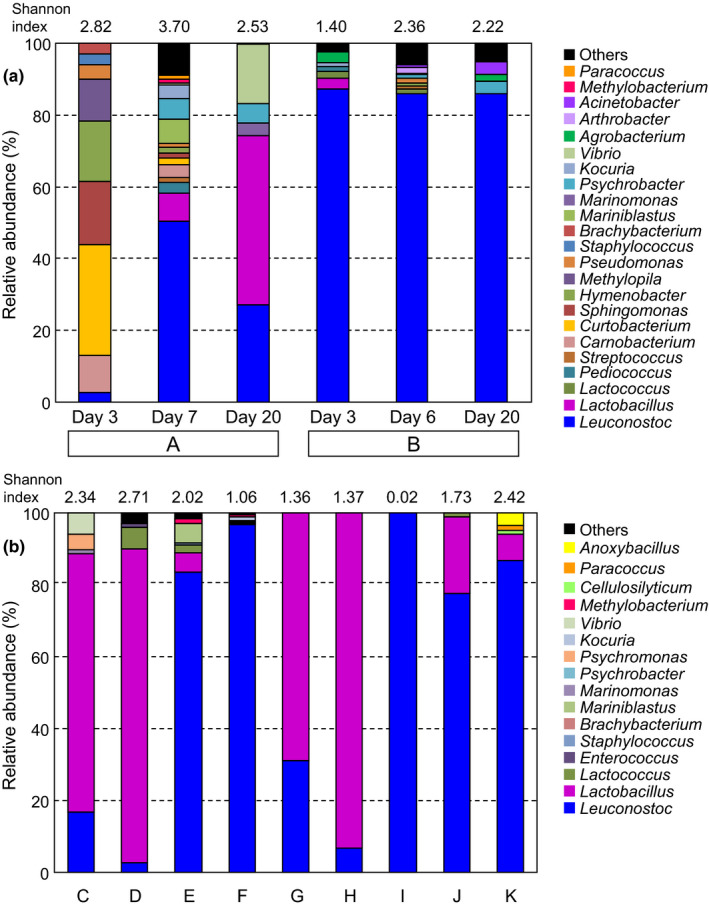
Relative abundance of the bacterial genera in senmaizuke. The bacterial composition was analyzed (a) over the course of the pickling and ripening processes, and (b) at the day of purchase. Microorganisms accounting for less than 1% of the population were classified into “others.” The samples and time course for product A and B correspond to those in Figure 2a

Next‐generation sequencing was performed to elucidate the bacterial composition in senmaizuke (Figure 3 and Table S2). Figure 3a shows the change in microbial diversity during the pickling and ripening processes. In the fermented‐type product A, the proportion of LAB to total bacterial community was increased during those processes. *Leuconostoc* and *Carnobacterium* were present at day 3, and *Leuconostoc* was predominant at day 7, whereas *Lactobacillus* became predominant at day 20. In the vinegar‐type product B, *Leuconostoc* was the dominant genus at day 3, with a small population of *Lactobacillus*, *Lactococcus,* and *Pediococcus*, and the dominance was maintained until day 20. This vigorous proliferation of LAB during early stages may be associated with acidified pH at day 3 (Figure 2a). Regarding products A and B, the bacterial diversity was the highest at the end of the main pickling process and was higher in the fermented‐type product than in the vinegar‐type product owing to the presence of bacteria other than LAB. Certain bacterial genera were diminished during ripening (day 20). Halophilic (for example *Psychrobacter*) and psychrophilic (for example *Vibrio* and *Marinilactibacillus*) bacteria were found in both product types.

*Lactobacillus* and a small population of *Leuconostoc* were predominant in the fermented‐type product C; either *Lactobacillus* or *Leuconostoc* was predominant in the vinegar‐type products (Figure 3b). LAB accounted for more than 90% of the population in products F, G, H, I, and J. A relatively high bacterial diversity was found in fermented‐type product C and in the vinegar‐type products D and K.

### 3.4 Growth characteristics of LAB in senmaizuke

The growth of microorganisms including LAB is generally retarded at low pH and low‐temperature conditions. I investigated whether the repressed LAB growth in the vinegar‐type senmaizuke was attributed to low pH and low‐temperature conditions. Incubation of product B at 20°C did not stimulate LAB growth; however, yeast growth increased (Table 1). When the pH of product B was adjusted to a neutral condition, the growth of LAB recovered, indicating that acidification via vinegar may be an important factor affecting LAB growth in senmaizuke (Table 1).

**TABLE 1:** Growth of LAB and yeast in product B during preservation

Preserved condition		Preserved period (day)	Fold change of CFU	
Temp.	pH		LAB	Yeasts
4°C	pH4 (Original)	7	0.0221	24.8
20°C	pH4 (Original)	4	0.675	710
4°C	pH6	13	76,300	21.2

Note

CFUs on agar media were evaluated before and after preservation of the products at the indicated conditions. LAB and yeast were naturally occurring cells in senmaizuke.

**FIGURE 4:**
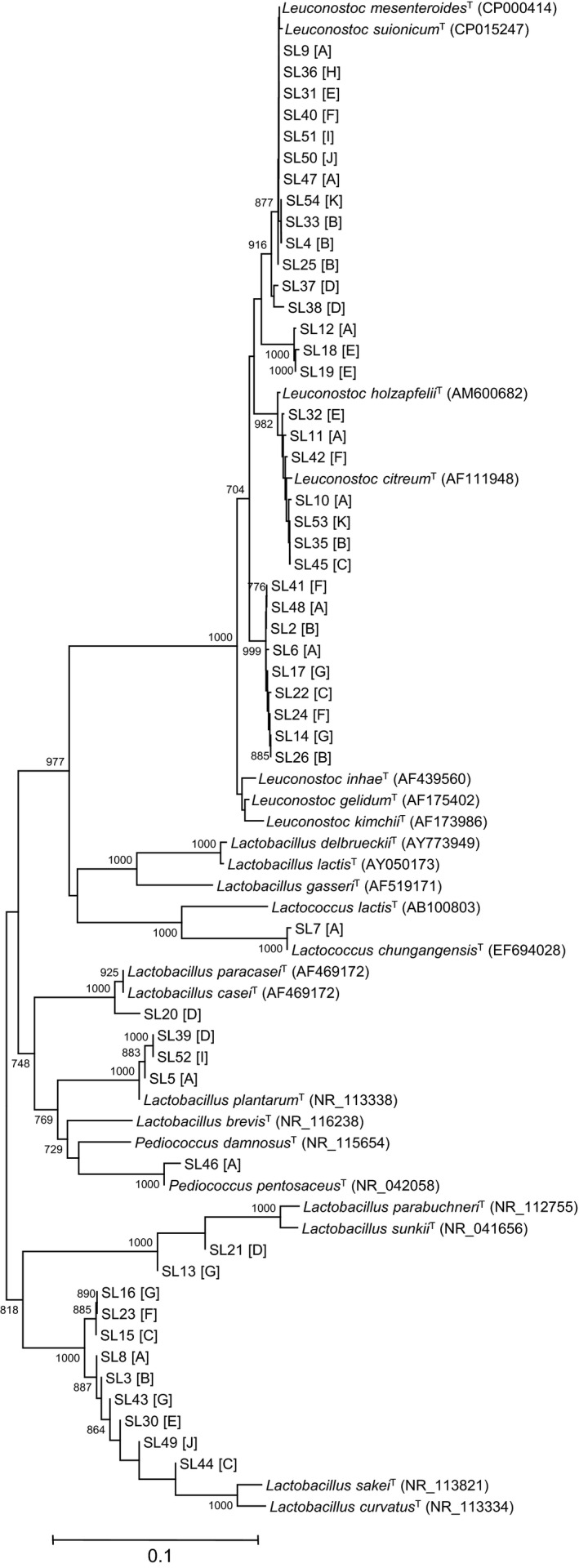
Phylogenetic relationships and diversity of LAB strains isolated from senmaizuke. The tree was constructed based on 16S rRNA gene sequences (ca. 650 bp) using the neighbor‐joining method. Bootstrap values ≥70% (for 1,000 replicates) are indicated at the branching points. Scale bar represents % estimated substitutions. The source of isolation (senmaizuke product) is shown in brackets. The GenBank accession numbers of type strains are shown in parentheses

Next, I isolated several LAB strains from senmaizuke products to examine their characteristics associated with growth in senmaizuke pickling conditions. Most isolates were phylogenetically classified as *Leu. mesenteroides, Leu. citreum, Leu. gelidum, Lb. sakei,* and *Lb. plantarum* (Figure 4). These species were also the predominant members identified via metagenomic sequencing (Table S2). I investigated whether the successive LAB growth observed in the fermented‐type senmaizuke was due to the enrichment of LAB strains possessing specific characteristics. Analysis of the *Lactobacillus* and *Leuconostoc* strains (15 and 28 strains, respectively) showed that their growth characteristics at low temperature (4°C) and low pH (pH4.7) were comparable between LAB isolated from the fermented‐type and vinegar‐type senmaizuke products (Table S3). *Lactobacillus* isolates showed higher growth than *Leuconostoc* isolates at low pH.

The carbon compounds present in senmaizuke were analyzed to identify the optimal carbon source for LAB growth during senmaizuke pickling. As the main carbon sources available for microbes in vegetables are known to be saccharides, the saccharide content in turnip juice was analyzed via HPLC (Figure S1). It was revealed that glucose (1.5%, w/v) and fructose (1.3%, w/v) were present in turnip juice at sufficient concentrations for enabling LAB growth, in addition to sucrose (0.10%, w/v) that is added as a raw material in senmaizuke pickling. These saccharides were utilized for growth by LAB isolates except for two strains (Table S3), which indicate that glucose and fructose derived from turnips, as well as sucrose added as a raw material, are important carbon sources for LAB growth during senmaizuke pickling.

### 3.5 Growth behavior of LAB and yeast isolates in the turnip medium

**FIGURE 5:**
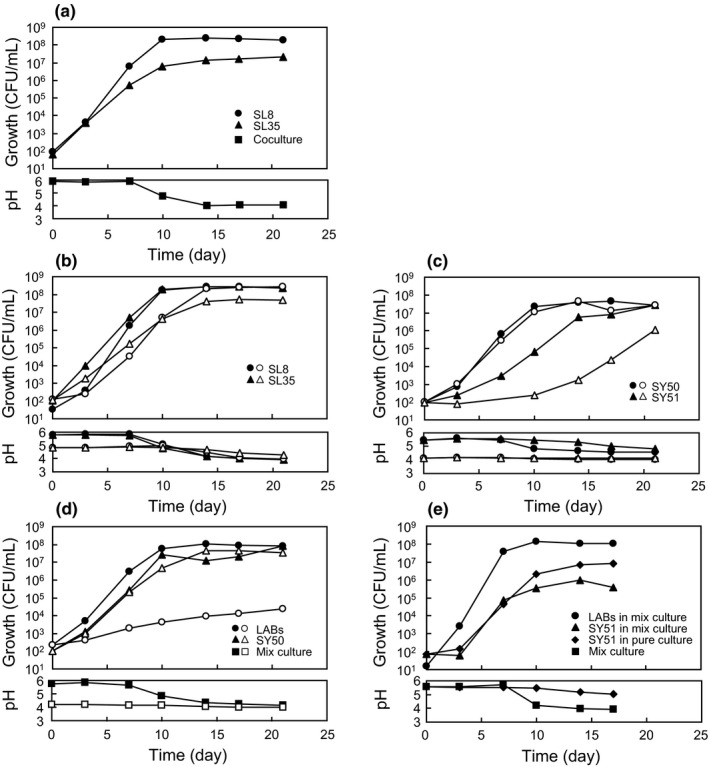
Growth of LAB and yeasts in a turnip medium simulating the senmaizuke environment. Initial pH of the medium was set at the original pH without adjustment (filled symbols) and at a low pH (open symbols). (a) Co‐culture comprising SL8 and SL35, (b) pure cultures of SL8 and SL35, (c) pure cultures of SY50 and SY51, (d) mixed culture comprising SL8, SL35, and SY50, (e) mixed culture comprising SL8, SL35 and SY51, and pure culture of SY51

A characteristic feature of the fermented‐type product A was the growth of *Lactobacillus* instead of *Leuconostoc*, and that of vinegar‐type product B was the growth of yeasts. The growth behavior was further evaluated by culturing the isolated strains in a turnip medium that mimicked the senmaizuke environment. In a co‐culture of *Lactobacillus* sp. SL8 and *Leuconostoc* sp. SL35, the final cell mass of SL8 was 10‐fold higher than that of SL35 (Figure 5a). This result was consistent with the LAB composition in product A at day 20 (Figure 3a). In pure cultures, SL8 and SL35 showed comparable final cell masses, whereas the final cell mass of SL35 alone reduced when a medium with relatively lower initial pH was used (Figure 5b). Factoring the change in pH in the medium, this reduced growth of SL35 in a co‐culture may be attributed to the sensitivity of SL35 to low pH. Moreover, SL8 exhibited a remarkable lag of growth until day 3 under initial low pH condition (Figure 5b), which indicated the weak adaptation ability of SL8 to the pickling environment.

Members of the yeast genera, *Debaryomyces* (representative strain SY51) and *Kazachstania* (representative strain SY50) were frequently isolated from the fermented‐type product A and the vinegar‐type product B, respectively. SY50 showed vigorous growth in pure culture and co‐culture with LAB, regardless of the initial pH of the medium (Figure 5c and 5d), indicating the ability to grow in vinegar‐type senmaizuke. SY51 was sensitive to low pH based on the slow growth in the pH4 medium (Figure 5c). In co‐cultures of SY51 and LAB, the retarded growth was observed at day 10 when acidification by LAB occurred (Figure 5e). These results indicate that the growth of SY51 was retarded in the presence of active LAB and vinegar.

## 4 DISCUSSION

The present study analyzed the microbial population, community, and growth in the traditional turnip pickle senmaizuke, and further evaluated the microbiological differences between the fermented‐type and vinegar‐type senmaizuke. A key characteristic feature determined was the successive growth of LAB during pickling and ripening processes in the fermented‐type senmaizuke (Figure 2a). The LAB population was as high as 10^8^ CFU/g (Figure 2, products A and C), which is a level at which pickles are defined as fermented pickles (Cho et al., 2006; Muller et al., 2018; Ogihara et al., 2009). After the ripening process, certain senmaizuke products contained a LAB community comprising *Leuconostoc* and *Lactobacillus* as well as halophilic bacteria such as *Vibrio* and psychrophilic bacteria such as *Psychrobacter* or *Psychromonas* (Figure 3, products A and C). During the manufacturing process, the microbial population in product A shifted from potentially soil‐ or plant‐derived bacteria such as *Curtobacterim* and *Sphingomonas* to LAB, and from *Leuconostoc* to *Lactobacillus* (Figure 3a) (Saddler et al., 2017; Yabuuchi & Kosako, 2015). These changes in the microbial community were attributed to the enrichment of LAB under favorable conditions of high salt, low temperature, and low oxygen concentration. The highest bacterial diversity and species richness at the end of the main pickling process was possibly the result of the addition of several raw materials in this step, which served as nutrients and a source of microbes (Figure 3a and Table S2). Characteristic features of the vinegar‐type senmaizuke included the steady growth of LAB after the preliminary pickling step (Figures 2 and 3a), and the bacterial community mainly comprising *Leuconostoc* and/or *Lactobacillus* (Figure 3). Since a large LAB population was present in the products (Figure 1a), most of the LAB community should be determined during the preliminary pickling in the vinegar‐type senmaizuke. Vinegar addition at the main pickling step is likely to repress subsequent LAB growth, and the experiment using neutral pH confirmed that the decrease of pH via vinegar addition repressed LAB growth (Table 1). The predominance of LAB in product B at the preliminary pickling step, which was earlier than that in product A, could be due to the lower salt concentration used (product B, 2.0%; product A, 5.1% in preliminary pickling). Product A requires addition of several salts for developing the flavor without the use of vinegar in pickling. In contrast to LAB profiles, the capability of successive yeast growth is also a feature of certain vinegar‐type senmaizuke products (Figure 2).

The microbial characteristics and pickling conditions required to generate these features of senmaizuke were investigated. Although LAB is phylogenetically diverse, members of two genera alone, *Leuconostoc* and *Lactobacillus*, were the major LAB present in senmaizuke products, in addition to *Lactococcus*, *Pediococcus*, *Carnobacterium*, *Streptococcus,* and *Enterococcus* present as transient members (Table S2). Phylogenetic analysis elucidated that senmaizuke products mainly contain *Leu. mesenteroides, Leu. citreum, Leu. gelidum, Lb. sakei* and *Lb. plantarum* (Figure 4 and Table S2). These *Leuconostoc* and *Lactobacillus* species have been detected in various vegetable pickles, and are thus considered as general members present in pickles (Swain et al., 2014; Tamang et al., 2016). The capability of growth at 4°C of several isolates derived from senmaizuke allowed the fermentation in the pickling process to be performed at low‐temperature conditions of winter season or refrigerator (Table S3). Moreover, their capabilities of utilizing glucose, fructose, and sucrose are advantageous for growth during senmaizuke pickling, which uses turnips and sucrose (Figure S1 and Table S3). The low pH tolerance of *Carnobacterium* and low sucrose utilization by *Pediococcus* (Franz et al., 2014; Pikuta & Hoover, 2014) were suspected to be the reason for which these members were outcompeted by other bacterial members during the pickling process. *Leuconostoc* is known to be more sensitive to acidity than *Lactobacillus* (Nieminen et al., 2014), which was similarly noted for the isolates derived from senmaizuke (Table S3). This difference of sensitivity has been reported to be associated with a change in microbial diversity from *Leuconostoc* to *Lactobacillus* members in the pickling process of kimchi, sauerkraut, and cucumber (Choi et al., 2003; Pederson, 1930; Singh & Ramesh, 2008). The result of microbial culturing explained the community shift in the fermented‐type senmaizuke (Figure 5): (1) the two genera could grow simultaneously, (2) *Leuconoctoc* stopped growing at a lower mass level than *Lactobacillus* when the medium was acidified, (3) *Lactobacillus* entered a lag phase for adapting to the pickling environment.

The adequate fermentation of pickles with microbes including LAB imparts desirable flavors; however, fermentation to a certain extent or with unsuitable microbial members leads to food spoilage. Yeast is often avoided in the pickle industry because of the production of undesirable gas, films, and odor (Hernandez et al., 2018). The risk of spoilage by yeast may be higher in the vinegar‐type senmaizuke than in the fermented‐type senmaizuke (Figure 1), and higher when the samples were stored at a higher room temperature (Table 1). *Kazachstania* showed the capability of growth in vinegar‐type senmaizuke containing low pH and in the presence of LAB (Figure 5). It is unclear why yeast growth, especially *Kazachstania,* was not observed in the fermented‐type senmaizuke, since *Kazachstania* members should be present in the factory environment. One possibility is that in comparison to the static bacterial activity present in vinegar‐type senmaizuke, the growth of several bacteria in fermentation‐type products resulted in microbial competition, thereby inhibiting yeast growth.

The findings of this study will elucidate the manufacturing fermentative process and improve the quality assurance of the traditional pickle senmaizuke. The role of each microbe in the flavor and taste of senmaizuke should be evaluated in future studies.

## CONFLICT OF INTEREST

The author declares that there is no conflict of interest.

## ETHICAL APPROVAL

This study does not involve any human or animal testing.

## Supporting information

Figure S1

Tables S1–S3

## Data Availability

The data that support the findings of this study are available from the corresponding author upon reasonable request.
