# Supplementary figures and images for: Microbial characterization of the Japanese traditional pickle senmaizuke produced by two different manufacturing processes
Source: Food Sci Nutr. 2021 Jun 19;9(8):4452–60. doi: 10.1002/fsn3.2419 (PMC8358362; doi:10.1002/fsn3.2419)

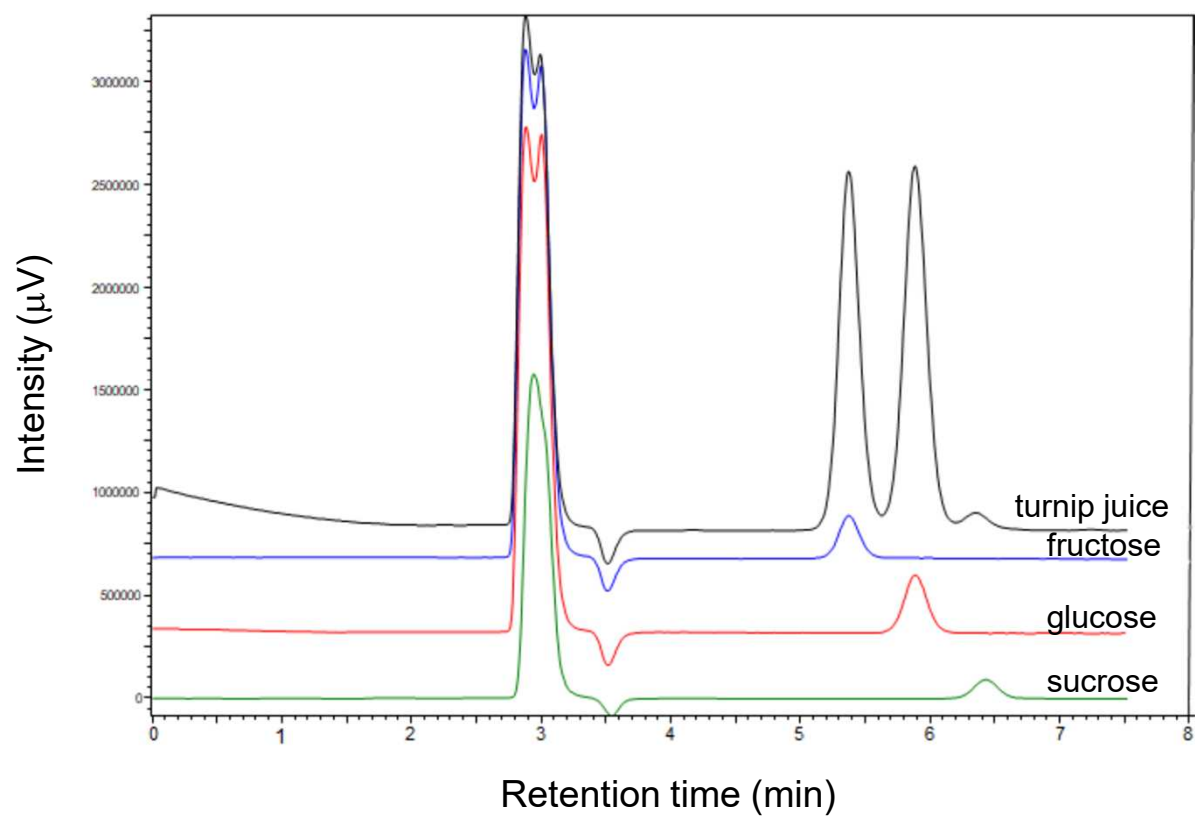

Figure S1

Supplement: Supplementary file 1 — Figure S1 [file FSN3-9-4452-s002.pdf]
